# Supplementary material for: Human farnesyl pyrophosphate synthase is allosterically inhibited by its own product
Source: Nat Commun. 2017 Jan 18;8:14132. doi: 10.1038/ncomms14132 (PMC5253651; doi:10.1038/ncomms14132)
Supplement: Supplementary Information — Supplementary figures. [file ncomms14132-s1.pdf]

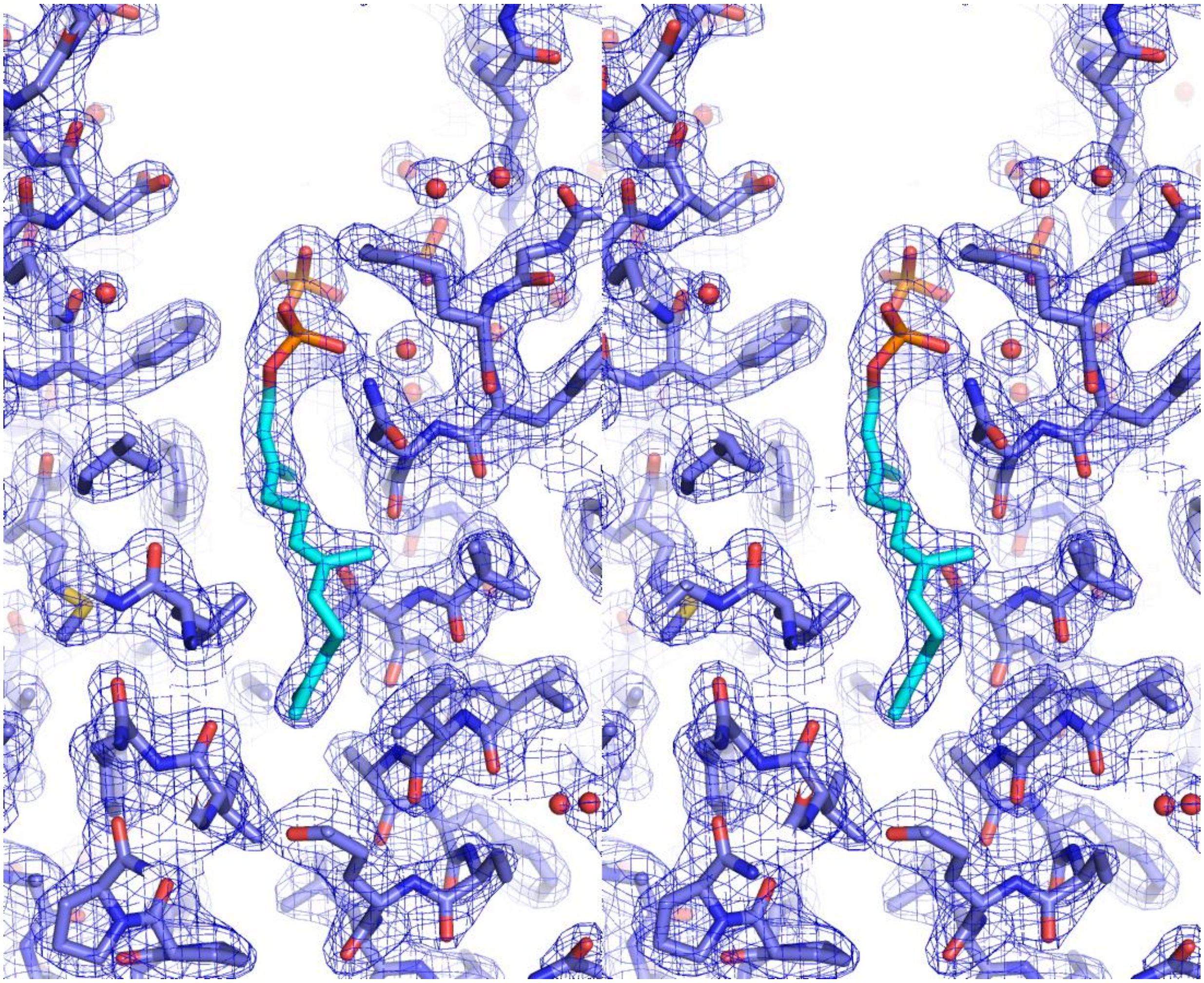

Supplementary Figure 1. Stereo view of electron density around FPP. Blue meshes represent the $2F_o$-F_c map (contoured at 1.0 σ) calculated with the final model. The bound ligand is shown in cyan, and the protein in blue.

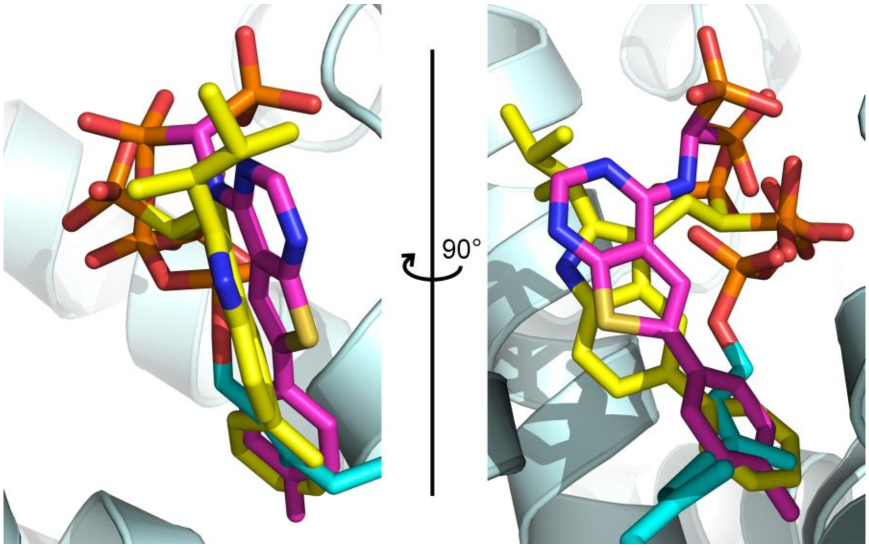

Supplementary Figure 2. Superimposition of allosterically bound FPP and bisphosphonate inhibitors. The perspective in the left panel is the same as in Fig. 2b. Inhibitors CL01131 (PDB ID 4LPG) and WC01088 (PDB ID 4QXS) are shown in magenta and yellow, respectively.
